# Value of Neighborhood Socioeconomic Status in Predicting Risk of Outcomes in Studies That Use Electronic Health Record Data

**DOI:** 10.1001/jamanetworkopen.2018.2716

**Published:** 2018-09-21

**Authors:** Nrupen A. Bhavsar, Aijing Gao, Matthew Phelan, Neha J. Pagidipati, Benjamin A. Goldstein

**Affiliations:** 1 Division of General Internal Medicine, Duke University School of Medicine, Durham, North Carolina; 2 Department of Biostatistics and Bioinformatics, Duke University School of Medicine, Durham, North Carolina; 3 Center for Predictive Medicine, Duke Clinical Research Institute, Durham, North Carolina; 4 Division of Cardiology, Duke University School of Medicine, Durham, North Carolina; 5 Children’s Health & Discovery Initiative, Duke University, Durham, North Carolina

## Abstract

**Importance:**

Data from electronic health records (EHRs) are increasingly used for risk prediction. However, EHRs do not reliably collect sociodemographic and neighborhood information, which has been shown to be associated with health. The added contribution of neighborhood socioeconomic status (nSES) in predicting health events is unknown and may help inform population-level risk reduction strategies.

**Objective:**

To quantify the association of nSES with adverse outcomes and the value of nSES in predicting the risk of adverse outcomes in EHR-based risk models.

**Design, Setting, and Participants:**

Cohort study in which data from 90 097 patients 18 years or older in the Duke University Health System and Lincoln Community Health Center EHR from January 1, 2009, to December 31, 2015, with at least 1 health care encounter and residence in Durham County, North Carolina, in the year prior to the index date were linked with census tract data to quantify the association between nSES and the risk of adverse outcomes. Machine learning methods were used to develop risk models and determine how adding nSES to EHR data affects risk prediction. Neighborhood socioeconomic status was defined using the Agency for Healthcare Research and Quality SES index, a weighted measure of multiple indicators of neighborhood deprivation.

**Main Outcomes and Measures:**

Outcomes included use of health care services (emergency department and inpatient and outpatient encounters) and hospitalizations due to accidents, asthma, influenza, myocardial infarction, and stroke.

**Results:**

Among the 90 097 patients in the training set of the study (57 507 women and 32 590 men; mean [SD] age, 47.2 [17.7] years) and the 122 812 patients in the testing set of the study (75 517 women and 47 295 men; mean [SD] age, 46.2 [17.9] years), those living in neighborhoods with lower nSES had a shorter time to use of emergency department services and inpatient encounters, as well as a shorter time to hospitalizations due to accidents, asthma, influenza, myocardial infarction, and stroke. The predictive value of nSES varied by outcome of interest (C statistic ranged from 0.50 to 0.63). When added to EHR variables, nSES did not improve predictive performance for any health outcome.

**Conclusions and Relevance:**

Social determinants of health, including nSES, are associated with the health of a patient. However, the results of this study suggest that information on nSES may not contribute much more to risk prediction above and beyond what is already provided by EHR data. Although this result does not mean that integrating social determinants of health into the EHR has no benefit, researchers may be able to use EHR data alone for population risk assessment.

## Key Points

**Question:**

What is the added predictive value of neighborhood socioeconomic status when predicting health outcomes and use of health care services with data from the electronic health record?

**Findings:**

In this cohort study, the predictive value of neighborhood socioeconomic status varied by outcome of interest. When added to electronic health record variables, neighborhood socioeconomic status did not improve predictive performance for any outcome.

**Meaning:**

These results suggest that information about the neighborhood in which a person lives may not contribute much more to risk prediction than information already within electronic health record data.

## Teaser

This cohort study quantifies the association of neighborhood socioeconomic status with adverse outcomes and the value of neighborhood socioeconomic status in predicting the risk of adverse outcomes in electronic health record (EHR)–based risk models.

## Introduction

Electronic health records (EHRs) have become an important component of clinical practice. However, a key limitation of EHRs when used for research purposes is that they do not reliably collect sociodemographic and neighborhood information, which has long been recognized to be strongly associated with health.^1^ Social and behavior measures linked to clinical variables within EHRs may improve clinical care and population health while also helping to inform population-level risk reduction strategies.^2^

Data from EHRs have been used extensively to develop risk models.^3^ Several studies have shown that linking neighborhood socioeconomic status (nSES) indicators with disease risk factors improves the accuracy of models in predicting disease outcomes.^4,5^ For instance, adding nSES indicators improves the accuracy of the Framingham risk score in the estimation of coronary heart disease risk.^6,7^ To our knowledge, there are few systematic studies assessing the value of nSES indicators in the prediction of diverse clinical events. In the present study, we supplemented individual EHR data with nSES data from the American Community Survey (ACS). We emphasize that our goal is not to assess whether nSES is associated with health outcomes—it undoubtedly is^8^—but to assess whether knowledge of nSES improves the prediction of health outcomes. Specifically, we sought to determine whether census tract–level nSES indicators are associated with poor health outcomes, whether census tract–level nSES data alone or in concert with EHR data can improve risk prediction beyond current models by using EHR data, and which elements in EHR indicators can serve as proxies for census tract–level nSES measures.

## Methods

### Clinical Data

Clinical data were derived from the EHR system of Duke University Health System (DUHS), which consists of 2 community hospitals, 1 large referral hospital, and a network of outpatient clinics. It is estimated that 85% of the residents of Durham County, North Carolina, receive their primary care from DUHS.^9^ We developed a data mart consisting of local patients by selecting those with an address in Durham County between January 1, 2009, and December 31, 2015, following the Patient-Centered Clinical Research Network Common Data Model, version 3.0, and adding custom fields, such as address and insurance status.^10^ We supplemented these data with EHR records from the Lincoln Community Health Center, a federally qualified health care facility serving a primarily underserved population in Durham County. All of the patients from the Lincoln Community Health Center were Durham residents. This study was approved by the Duke University School of Medicine institutional review board, which also granted a waiver of informed consent for this study because this is a secondary data analysis. This study followed the Strengthening the Reporting of Observational Studies in Epidemiology (STROBE) reporting guideline.

#### Sample

We divided our cohort into training and testing sets. The index date (ie, time zero) for the training set was January 1, 2009, and the index date for the testing set was January 1, 2012. To be eligible at the index time point, patients had to be age 18 years or older, have at least 1 health care encounter in the year prior to the index date, and be a Durham County resident at their last encounter. This protocol allowed us to characterize local patients who were actively seeking care at DUHS. The data mart contained encounter data through December 31, 2016.

#### Clinical Outcomes

We chose a broad range of outcomes based on the use of services (emergency department and inpatient and outpatient encounters) and hospitalizations due to accidents, asthma, influenza, myocardial infarction, and stroke. These clinical outcomes were chosen for their known association with nSES.^11,12,13,14,15,16,17^ Cause-specific hospitalizations were defined via discharge diagnosis. Patients were censored at their last encounter date in the data mart or 3 years after the index date (December 31, 2011, for the training set; December 31, 2015, for the testing set), whichever came first. Because patients had potential follow-up through the end of 2016, we had a “burnout” period when we could properly capture the censoring date.^18^

#### Clinical Predictors

We abstracted 41 baseline predictors from our data mart that are commonly available in EHR systems, including measures of demographics, comorbidities, laboratory tests, medications, and use of health care services (eTable 1 in the Supplement). We used encounter data from the year prior to the index dates (ie, 2008 for the training set and 2011 for the testing set) to define predictor values. We presumed that the absence of a measurement (eg, no *International Classification of Diseases, Ninth Revision* code for diabetes) indicated that the individual did not have the condition. Because not all patients had all laboratory tests performed, instead of imputing missing values, we simply used the number of times the test was administered, a metric that has been shown to be predictive of outcomes.^19^

### nSES Data

To define nSES, we extracted data from the 2010 ACS. The ACS is a rolling survey of the US population that gathers information, such as ancestry, educational level, income, language proficiency, migration, disability, employment, and housing characteristics, across 1298 variables.^20^ The ACS releases estimates at the regional, state, and county level every year, and data at the census-tract and block-group levels are available every 5 years. For our study, a patient’s address at the index date was used to identify their census tract. Census tracts are small geographical units of approximately 4000 residents. Durham County has 73 census tracts. To calculate nSES, we used the Agency for Healthcare Research and Quality (AHRQ) SES index.^21^ The index is a weighted combination of the percentage of households with a mean number of 1 person or more per room, the median value of owner-occupied dwelling, the percentage unemployed, percentage living below the poverty level, the median household income, the percentage 25 years or older with a bachelor’s degree or higher, and the percentage 25 years or older with less than a 12th-grade education. It is scaled to the US population to lie between 0 and 100, with a higher number indicative of greater neighborhood deprivation. Previous studies have used this index to represent a geographical area–based measure of the socioeconomic deprivation experienced according to neighborhood.^22,23,24,25^

### Statistical Analysis

The characteristics of the patients were summarized by county-level quartiles of the nSES score. Categorical variables were presented as frequencies, and continuous variables were presented as mean (SD) values. We assessed the amount of variation within nSES explained by EHR data by regressing nSES onto all the EHR data and calculating *R*^2^ statistics. To evaluate the differences in time to events based on nSES, we fit Kaplan-Meier curves stratified on quartiles of nSES. We assessed differences via a log-rank test. We next tried to determine how adding nSES to the EHR data affects risk prediction. To derive our prediction model, we used random survival forest (RSF).^26^ The random forests method is an extension of classification and regression trees, which combines multiple trees via a process called *bagging* (bootstrap aggregation) to create a more robust predictor.^27,28^ The RSF is an application of random forests to time-to-event data. In brief, RSF (and random forests) provides a nonparametric means of developing predictive models. Its primary value is that it allows one to model both nonlinear and heterogeneous (interaction) effects. This is a more robust model than the standard Cox proportional hazards regression model. Using the training data, we first trained an RSF model using only the EHR data. Next, we fit a second model including nSES as an additional predictor. We used the test data to assess the predictive performance of both models. We calculated C statistics appropriate for time-to-event data and compared them using the permutation test.^29,30^ All *P* values were from 2-sided tests, and results were deemed statistically significant at *P* < .05. The C statistic, also termed *concordance statistic* or *c-index*, is analogous to the area under the curve and is a global measure of model discrimination.^31^ Discrimination refers to the ability of a risk prediction model to separate patients who develop a health outcome from patients who do not develop a health outcome.^31^ Effectively, the C statistic is the probability that a model will result in a higher-risk score for a patient who develops the outcomes of interest compared with a patient who does not develop the outcomes of interest.

### Sensitivity Analysis

For sensitivity analysis, we used cross-validation within the training set based on the RSF to assess the added value of nSES. We also used a more general parameterization of the ACS variables. We performed a principle components analysis and selected the components that explained at least 95% of the variance. All statistical analyses were performed in R, version 3.1.4 (The R Foundation for Statistical Computing). We used the package randomForestSRC to build the RSF model, and we used the package survAUC to calculate the C statistic.^32,33^

## Results

### Descriptive Measures

**Figure 1.**
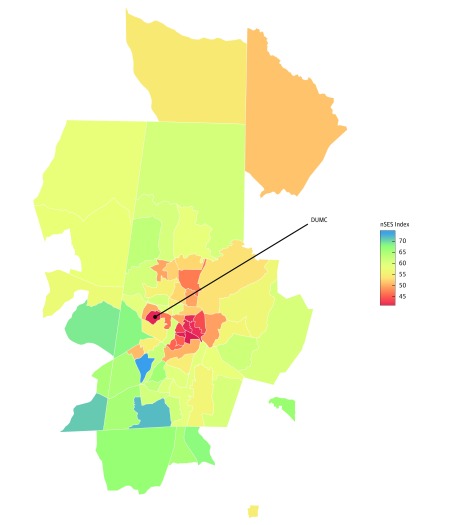
Heatmap of Neighborhood Socioeconomic Status (nSES) by Census Tract in Durham County, North Carolina, Based on 2010 American Community Survey Estimates Duke University Medical Center (DUMC) is located in central Durham County. Redder colors indicate poorer nSES, while bluer colors indicate better nSES. The northern parts of the county are fairly rural, while the center parts, where nSES is lower, are more urban.

We identified 90 097 eligible patients for the training data and 122 812 eligible patients for the test data. The demographics and clinical characteristics of these patients, stratified by nSES quartiles and training or test set, are shown in eTable 1 in the Supplement, with a reduced set of demographics in Table 1. The population in the training data set was predominately female (57 507 [63.8%]) and black (37 774 [41.9%]), with a mean (SD) age of 47.2 (17.7) years. Similar characteristics were seen in the testing data set (75 517 [61.5%] female; 48 766 [39.7%] black; mean [SD] age, 46.2 [17.9] years). Patients living in neighborhoods in a lower nSES quartile were more likely to be younger, black, have public insurance, and experience more clinical health care encounters than those in a higher nSES quartile. Clinically, those in a lower nSES quartile were also more likely to have more comorbidities, take more medications, and undergo more laboratory tests. Figure 1 displays the spatial distribution of nSES across 73 census tracts of Durham County. Overall, nSES ranged from a scaled value of 37% to 74%. The northern parts of Durham County are quite rural, and the central parts are fairly urban.

**Table 1.** Reduced Demographic Table for Training and Testing Set

Characteristic	Overall^a^		Socioeconomic Status^a^			
	Training Set (n = 90 097)	Testing Set (n = 122 812)	Quartile 1 (n = 21 906)	Quartile 2 (n = 21 960)	Quartile 3 (n = 25 071)	Quartile 4 (n = 21 160)
Male sex	32 590 (36.2)	47 295 (38.5)	7779 (35.5)	7561 (34.4)	9213 (36.7)	8037 (38.0)
Age, mean (SD), y	47.2 (17.7)	46.2 (17.9)	44.8 (18.0)	46.4 (17.9)	48.0 (17.6)	49.4 (17.1)
Race/ethnicity						
Black	37 774 (41.9)	48 766 (39.7)	16 351 (74.6)	10 624 (48.4)	6417 (25.6)	4382 (20.7)
Hispanic	3831 (4.3)	7528 (6.1)	1478 (6.7)	1430 (6.5)	600 (2.4)	323 (1.5)
White	5542 (6.2)	10 197 (8.3)	3086 (14.1)	8686 (39.6)	16 400 (65.4)	14 778 (69.8)
Other	42 950 (47.7)	56 321 (45.9)	991 (4.5)	1220 (5.6)	1654 (6.6)	1677 (7.9)
Primary payer						
Private	53 326 (59.2)	68 473 (55.8)	7795 (35.6)	12 332 (56.2)	17 348 (69.2)	15 851 (74.9)
Public	21 566 (23.9)	28 032 (22.8)	6719 (30.7)	5422 (24.7)	5291 (21.1)	4134 (19.5)
Self-pay	7031 (7.8)	13 656 (11.1)	3203 (14.6)	1878 (8.6)	1249 (5.0)	701 (3.3)
Unknown	8174 (9.1)	12 651 (10.3)	4189 (19.1)	2328 (10.6)	1183 (4.7)	474 (2.2)
Use of health care services						
Emergency visits, mean (SD), d	0.48 (1.46)	0.44 (1.37)	0.97 (2.14)	0.52 (1.51)	0.27 (0.95)	0.16 (0.66)
Inpatient visits, mean (SD), d	0.14 (0.53)	0.11 (0.46)	0.20 (0.68)	0.14 (0.55)	0.11 (0.46)	0.09 (0.38)
Outpatient visits, mean (SD), d	6.37 (8.86)	7.07 (10.10)	6.59 (9.22)	6.42 (9.17)	6.21 (8.64)	6.31 (8.40)
Inpatient length of stay, mean (SD), d	0.64 (4.04)	0.54 (4.15)	0.98 (4.86)	0.69 (4.70)	0.51 (3.44)	0.40 (2.82)
Public clinic visit	11 603 (12.9)	18 160 (14.8)	6077 (27.7)	3336 (15.2)	1570 (6.3)	620 (2.9)

^a^ Data are presented as number (percentage) of patients unless otherwise indicated.

#### Association Between nSES and Health Outcomes

**Figure 2.**
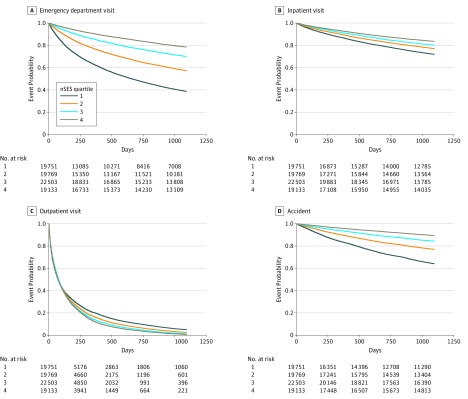
Kaplan-Meier Plots for Time to Emergency Department Visit (A), Inpatient Visit (B), Outpatient Visit (C), and Accident (D) Stratified on Quartiles of Neighborhood Socioeconomic Status (nSES) With the exception of outpatient visit, those in the lowest neighborhood quartiles have the quickest time to the event. Quartile 1 indicates lower nSES, while quartile 4 indicates better nSES. Those in areas with lower nSES have quicker time to events than those in areas with higher nSES.

**Figure 3.**
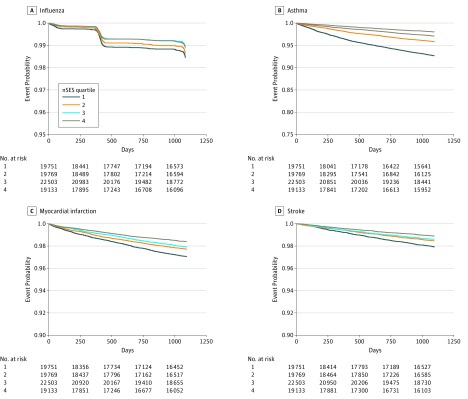
Kaplan-Meier Plots for Time to Influenza (A), Asthma Hospitalization (B), Myocardial Infarction (C), and Stroke (D) Stratified on Quartiles of Neighborhood Socioeconomic Status (nSES) While event rates are relatively low, those in the lowest neighborhood quartiles have the quickest time to the event. Quartile 1 indicates lower nSES, while quartile 4 indicates better nSES. Those in areas with lower nSES have quicker time to events than those in areas with higher nSES.

Next, we assessed differences in time-to-health outcomes based on nSES. Figure 2 and Figure 3 show Kaplan-Meier plots for the 8 different outcomes. (The eFigure in the Supplement provides risk set information.) The log-rank test was significant for all outcomes. In addition, for all outcomes, those in lower nSES neighborhoods had shorter times to events. The one exception was outpatient encounters; individuals in neighborhoods with a higher nSES had a shorter time to the next appointment.

#### Added Value of nSES for Risk Assessment

Finally, we assessed the added predictive value of nSES to clinical variables readily available in the EHR. Table 2 shows the C statistics for the 8 outcomes based on EHR data alone, nSES information alone, and EHR data and nSES information combined. The predictive value of nSES varies by different outcomes of interest. Although nSES was moderately predictive for most outcomes (C statistic ranged from 0.50 to 0.63), it did not improve predictive performance for any outcome when added to EHR variables.

**Table 2.** C Statistics of Different Outcomes for Different Predictor Sets

Outcome	C Statistics		
	EHR	EHR and nSES	nSES
Myocardial infarction	0.892	0.892	0.525
Stroke	0.854	0.855	0.500
Asthma	0.752	0.756	0.608
Accident	0.747	0.755	0.606
Emergency department visit	0.746	0.752	0.629
Inpatient visit	0.740	0.742	0.532
Outpatient visit	0.674	0.677	0.507
Influenza	0.562	0.565	0.503

Abbreviations: EHR, electronic health record; nSES, neighborhood socioeconomic status.

To understand the lack of added predictive value better, we regressed nSES onto the EHR variables, estimating the coefficient of determination (*R*^2^). All EHR data explained 31.2% of the variability in nSES, while demographic factors alone (age, sex, race/ethnicity, and insurance status) explained 28.7% of the variance, suggesting that a moderate amount of the variation in nSES is explained by demographic factors alone.

### Sensitivity Analysis

In our sensitivity analysis, both the use of the estimate based on the RSF within the training data and principal components to represent ACS data provided similar results (eTables 2 and 3 in the Supplement). We also hypothesized that nSES information would be more predictive for long-term outcomes compared with short-term outcomes. When we examined the added value of nSES for 30-day, 90-day, 180-day, 1-year, 2-year, and 3-year time horizons, we found that nSES did not improve prediction over longer-term horizons (eFigure in the Supplement).

## Discussion

Our study found that, while the risk of clinical outcomes differs based on nSES, and although nSES is moderately predictive of clinical outcomes, nSES does not meaningfully improve risk prediction of clinical events above and beyond what is easily extractable from the EHR. A primary explanation for this finding could be that, at least in our population, demographic characteristics are highly associated with nSES. In our study, knowledge of a patient’s age, sex, race/ethnicity, and insurance status explained more than 28% of the variability in nSES. For comparison, it is typical for the coefficient of determination to be less than 10% in clinical studies. To our knowledge, this study is one of the first to broadly assess the added value of nSES in a large, population-based risk prediction study using data from the EHR.

### EHRs and Population Health

There has been increasing emphasis on the use of data from the EHR for population health.^34^ There is potential to use these data to understand the health of communities through activities such as disease surveillance and population risk assessment, especially when medical centers, such as DUHS, are the primary health care facility in a community.^35^ This use is increasingly salient amid changes to patient reimbursement in which medical centers are becoming financially responsible for managing the health of their patient populations.^36^ One of the concerns with EHR data are that they lack important contextual information regarding patients’ social environments.^37^ To this end, widely available nSES data may be linked to patients’ EHRs.

The goal of identifying neighborhoods with greater health care needs is to deploy pragmatic interventions, such as patient navigators, social workers, or access to telemedicine, which can target high-risk populations. To quantify nSES, we used data available from the ACS to calculate the AHRQ nSES index. Others have used the AHRQ nSES index to assess outcomes, such as prevalence of chronic disease and risk of hospital readmission, and, similar to our study, they found that lower nSES was associated with poorer health outcomes.^23,24^ In our study, we explored the effect that different measures of nSES may have on our results through a sensitivity analysis that used principal components analysis, which was conducted on all variables present in the ACS data set to identify constructs that may have better discriminatory characteristics than the AHRQ risk score alone. We did not see any appreciable differences in C statistics when we used the principal components analysis–derived constructs compared with the AHRQ risk score.

### Neighborhoods and Health

It is well known that neighborhoods are significantly associated with the health of their residents through physical and social attributes.^8^ The mechanisms by which neighborhoods are associated with health include increased stress level, decreased physical activity, and poor nutrition, which in turn affect both proximal risk factors, such as blood pressure, diabetes control, and inflammation, and distal health outcomes, such as cardiovascular disease.^8^ The democratization of neighborhood-level contextual data, the ability to link these data to the EHR, and the ability to target population-level interventions to high-risk areas have resulted in a resurgence in research related to neighborhoods and health. Our results support prior research in this area by showing that patients who live in areas with lower nSES have poorer health outcomes than patients who live in areas with higher nSES.^38,39,40^ As an extension of this finding, we examined the importance of nSES in risk prediction across multiple health and service-use outcomes and found little added value for the risk prediction models within our population. This area of research has not been extensively studied; however, prior studies may help place our results in context. Fiscella and colleagues^41^ showed that adding individual-level nSES (ie, educational level and income) to the Framingham risk score improved calibration of the risk model for coronary heart disease, but not discrimination, while reducing bias in risk prediction for coronary heart disease for those with lower socioeconomic status. They did not use nSES measures.

In a separate study of 1178 consecutive patients 65 years or younger who were discharged from 8 hospitals in central Israel, the C statistic for predicting mortality after myocardial infarction significantly improved from 0.72 to 0.76 (*P* < .001) after socioeconomic status measures, including nSES, were added to the basic prediction model. The study used an index developed by the Israel Central Bureau of Statistics, which may not be generalizable to other populations, and the extended model included both individual-level socioeconomic status and nSES predictors.^42^

In a study of 109 793 patients from the Cleveland Clinic Health System, Dalton and colleagues^43^ showed that the pooled cohort equation risk model predicted events associated with atherosclerotic cardiovascular disease with greater discrimination among individuals living in more affluent communities, as defined using the neighborhood disadvantage index, than among individuals living in poorer neighborhoods. These results may suggest that the predictive ability of nSES might depend on the nSES index used and the population within which it is applied.

### Strengths and Limitations

This study has some strengths. Durham County is a diverse county with both wealthy and poor residents as well as both urban and rural neighborhoods. We were able to use our large sample size and relatively long follow-up to quantify outcomes with low event rates. There are also important limitations to our study. These clinical data are from one geographical region, and it is possible that, in a region with different demographic characteristics, the *R*^2^ would be lower, allowing for greater contribution of nSES in risk prediction. However, insurance status alone had an *R*^2^ of 12.5%. In addition, our models were developed and validated using EHR data from a single institution (DUHS and Lincoln Community Health Center share a common EHR system). Patients who received care at different institutions would be missed. We also do not have data on health care received outside DUHS or Lincoln Community Health Center by the patients included in our study. In addition, we used only 1 primary parameterization of nSES: the ARHQ neighborhood deprivation index. It is possible that other measures, such as the Gini index, would have yielded greater added value.^44^ That being said, our more agnostic principal components analysis yielded similar results. Finally, although RSF is a robust model algorithm capable of finding complex effects, it is possible that another modeling approach would have yielded different results.^45^

## Conclusions

This work reaffirms that the social environment is associated with health outcomes. However, these results suggest that information about the environment in which a person lives may not contribute much more to population risk assessment than is already provided by EHR data. Although this result does not mean that integrating social determinants of health into the EHR has no benefit, researchers may be able to use EHR data alone for population risk assessment.

## Notes

Supplement. **eTable 1.** Demographic and Clinical Characteristics of Patients **eTable 2.** Model Fit Results Using Cross-Validation in the Training Data **eTable 3.** Model Fit Results Using Principal Components of ACS Data **eFigure.** Discrete Time Prediction Results

## References

[1] Galiatsatos P, Kineza C, Hwang S, Neighbourhood characteristics and health outcomes: evaluating the association between socioeconomic status, tobacco store density and health outcomes in Baltimore City. Tob Control. 2018;27(e1):-. doi:10.1136/tobaccocontrol-2017-053945 29170167 PMC5966324

[2] Casey JA, Schwartz BS, Stewart WF, Adler NE Using electronic health records for population health research: a review of methods and applications. Annu Rev Public Health. 2016;37:61-81. doi:10.1146/annurev-publhealth-032315-021353 26667605 PMC6724703

[3] Goldstein BA, Navar AM, Pencina MJ, Ioannidis JP Opportunities and challenges in developing risk prediction models with electronic health records data: a systematic review. J Am Med Inform Assoc. 2017;24(1):198-208. doi:10.1093/jamia/ocw042 27189013 PMC5201180

[4] Chen C, Weider K, Konopka K, Danis M Incorporation of socioeconomic status indicators into policies for the meaningful use of electronic health records. J Health Care Poor Underserved. 2014;25(1):1-16. doi:10.1353/hpu.2014.0040 24509007 PMC3951370

[5] Steenland K, Henley J, Calle E, Thun M Individual- and area-level socioeconomic status variables as predictors of mortality in a cohort of 179,383 persons. Am J Epidemiol. 2004;159(11):1047-1056. doi:10.1093/aje/kwh129 15155289

[6] Brindle PM, McConnachie A, Upton MN, Hart CL, Davey Smith G, Watt GC The accuracy of the Framingham risk-score in different socioeconomic groups: a prospective study. Br J Gen Pract. 2005;55(520):838-845. 16281999 PMC1570792

[7] Franks P, Tancredi DJ, Winters P, Fiscella K Including socioeconomic status in coronary heart disease risk estimation. Ann Fam Med. 2010;8(5):447-453. doi:10.1370/afm.1167 20843887 PMC2939421

[8] Diez Roux AV, Mair C Neighborhoods and health. Ann N Y Acad Sci. 2010;1186:125-145. doi:10.1111/j.1749-6632.2009.05333.x 20201871

[9] Miranda ML, Ferranti J, Strauss B, Neelon B, Califf RM Geographic health information systems: a platform to support the ‘triple aim’. Health Aff (Millwood). 2013;32(9):1608-1615. doi:10.1377/hlthaff.2012.1199 24019366 PMC4076782

[10] Corley DA, Feigelson HS, Lieu TA, McGlynn EA Building data infrastructure to evaluate and improve quality: PCORnet. J Oncol Pract. 2015;11(3):204-206. doi:10.1200/JOP.2014.003194 25980016 PMC4438109

[11] Chandrasekhar R, Sloan C, Mitchel E, Social determinants of influenza hospitalization in the United States. Influenza Other Respir Viruses. 2017;11(6):479-488. doi:10.1111/irv.12483 28872776 PMC5720587

[12] Claudio L, Tulton L, Doucette J, Landrigan PJ Socioeconomic factors and asthma hospitalization rates in New York City. J Asthma. 1999;36(4):343-350. doi:10.3109/02770909909068227 10386498

[13] Foraker RE, Patel MD, Whitsel EA, Suchindran CM, Heiss G, Rose KM Neighborhood socioeconomic disparities and 1-year case fatality after incident myocardial infarction: the Atherosclerosis Risk in Communities (ARIC) Community Surveillance (1992-2002). Am Heart J. 2013;165(1):102-107. doi:10.1016/j.ahj.2012.10.022 23237140 PMC3523273

[14] Gerber Y, Koton S, Goldbourt U, ; Israel Study Group on First Acute Myocardial Infarction Poor neighborhood socioeconomic status and risk of ischemic stroke after myocardial infarction. Epidemiology. 2011;22(2):162-169. doi:10.1097/EDE.0b013e31820463a3 21131822

[15] Koopman C, van Oeffelen AA, Bots ML, Neighbourhood socioeconomic inequalities in incidence of acute myocardial infarction: a cohort study quantifying age- and gender-specific differences in relative and absolute terms. BMC Public Health. 2012;12:617. doi:10.1186/1471-2458-12-617 22870916 PMC3490806

[16] Lawson F, Schuurman N, Amram O, Nathens AB A geospatial analysis of the relationship between neighbourhood socioeconomic status and adult severe injury in Greater Vancouver. Inj Prev. 2015;21(4):260-265. doi:10.1136/injuryprev-2014-041437 25694418 PMC4518736

[17] Zarzaur BL, Croce MA, Fabian TC, Fischer P, Magnotti LJ A population-based analysis of neighborhood socioeconomic status and injury admission rates and in-hospital mortality. J Am Coll Surg. 2010;211(2):216-223. doi:10.1016/j.jamcollsurg.2010.03.036 20670859 PMC3042251

[18] Phelan M, Bhavsar NA, Goldstein BA Illustrating informed presence bias in electronic health records data: how patient interactions with a health system can impact inference. EGEMS (Wash DC). 2017;5(1):22. doi:10.5334/egems.243 29930963 PMC5994954

[19] Goldstein BA, Pomann GM, Winkelmayer WC, Pencina MJ A comparison of risk prediction methods using repeated observations: an application to electronic health records for hemodialysis. Stat Med. 2017;36(17):2750-2763. doi:10.1002/sim.7308 28464332 PMC5494276

[20] Alexander CA Still rolling: Leslie Kish’s ‘rolling samples’ and the American Community Survey. Surv Methodol. 2002;28(1):35-41.

[21] Bonito AJ, Bann C, Eicheldinger C, Carpenter L Creation of new race-ethnicity codes and socioeconomic status (SES) indicators for Medicare beneficiaries: final report, sub-task 21. Rockville, MD: Agency for Healthcare Research and Quality; 2008. AHRQ Publication 08-0029-EF.

[22] Berkowitz SA, Traore CY, Singer DE, Atlas SJ Evaluating area-based socioeconomic status indicators for monitoring disparities within health care systems: results from a primary care network. Health Serv Res. 2015;50(2):398-417. doi:10.1111/1475-6773.12229 25219917 PMC4369215

[23] Billings J, Zeitel L, Lukomnik J, Carey TS, Blank AE, Newman L Impact of socioeconomic status on hospital use in New York City. Health Aff (Millwood). 1993;12(1):162-173. doi:10.1377/hlthaff.12.1.162 8509018

[24] Lang IA, Llewellyn DJ, Langa KM, Wallace RB, Huppert FA, Melzer D Neighborhood deprivation, individual socioeconomic status, and cognitive function in older people: analyses from the English Longitudinal Study of Ageing. J Am Geriatr Soc. 2008;56(2):191-198. doi:10.1111/j.1532-5415.2007.01557.x 18179489 PMC2671806

[25] Putnam LR, Tsao K, Nguyen HT, Kellagher CM, Lally KP, Austin MT The impact of socioeconomic status on appendiceal perforation in pediatric appendicitis. J Pediatr. 2016;170:156-160. doi:10.1016/j.jpeds.2015.11.075 26922766

[26] Ishwaran H, Kogalur UB, Blackstone EH, Lauer MS Random survival forests. Ann Appl Stat. 2008;2(3):841-860. doi:10.1214/08-AOAS169

[27] Breiman L Random forests. Mach Learn. 2001;45(1):5-32. doi:10.1023/A:1010933404324

[28] Breiman L, Friedman J, Stone CJ, Olshen RA Classification and Regression Trees. Boca Raton, FL: Chapman and Hall/CRC; 1984.

[29] Uno H, Cai T, Pencina MJ, D’Agostino RB, Wei LJ On the C-statistics for evaluating overall adequacy of risk prediction procedures with censored survival data. Stat Med. 2011;30(10):1105-1117. doi:10.1002/sim.4154 21484848 PMC3079915

[30] Venkatraman ES A permutation test to compare receiver operating characteristic curves. Biometrics. 2000;56(4):1134-1138. doi:10.1111/j.0006-341X.2000.01134.x 11129471

[31] Pencina MJ, D’Agostino RB Sr Evaluating discrimination of risk prediction models: the C statistic. JAMA. 2015;314(10):1063-1064. doi:10.1001/jama.2015.11082 26348755

[32] Ishwaran H, Kogalur UB Random survival forests for R. R News. 2007;7(2):25-31.

[33] Schmid M, Potapov S, Adler W survAUC: estimators of prediction accuracy for time-to-event data. Presented at: R User Conference; 2011; University of Warwick, Coventry, UK.

[34] Friedman DJ, Parrish RG, Ross DA Electronic health records and US public health: current realities and future promise. Am J Public Health. 2013;103(9):1560-1567. doi:10.2105/AJPH.2013.301220 23865646 PMC3780677

[35] McVeigh KH, Newton-Dame R, Chan PY, Can electronic health records be used for population health surveillance? validating population health metrics against established survey data. EGEMS (Wash DC). 2016;4(1):1267. doi:10.13063/2327-9214.1267 28154837 PMC5226379

[36] Clough JD, McClellan M Implementing MACRA: implications for physicians and for physician leadership. JAMA. 2016;315(22):2397-2398. doi:10.1001/jama.2016.7041 27213914

[37] Gold R, Cottrell E, Bunce A, Developing electronic health record (EHR) strategies related to health center patients’ social determinants of health. J Am Board Fam Med. 2017;30(4):428-447. doi:10.3122/jabfm.2017.04.170046 28720625 PMC5618800

[38] Nelson K, Schwartz G, Hernandez S, Simonetti J, Curtis I, Fihn SD The association between neighborhood environment and mortality: results from a national study of veterans. J Gen Intern Med. 2017;32(4):416-422. doi:10.1007/s11606-016-3905-x 27815763 PMC5377878

[39] Pollack CE, Slaughter ME, Griffin BA, Dubowitz T, Bird CE Neighborhood socioeconomic status and coronary heart disease risk prediction in a nationally representative sample. Public Health. 2012;126(10):827-835. doi:10.1016/j.puhe.2012.05.028 23083844 PMC3488146

[40] Wang L, Porter B, Maynard C, Predicting risk of hospitalization or death among patients receiving primary care in the Veterans Health Administration. Med Care. 2013;51(4):368-373. doi:10.1097/MLR.0b013e31827da95a 23269113

[41] Fiscella K, Tancredi D, Franks P Adding socioeconomic status to Framingham scoring to reduce disparities in coronary risk assessment. Am Heart J. 2009;157(6):988-994. doi:10.1016/j.ahj.2009.03.019 19464408

[42] Molshatzki N, Drory Y, Myers V, Role of socioeconomic status measures in long-term mortality risk prediction after myocardial infarction. Med Care. 2011;49(7):673-678. doi:10.1097/MLR.0b013e318222a508 21666512

[43] Dalton JE, Perzynski AT, Zidar DA, Accuracy of cardiovascular risk prediction varies by neighborhood socioeconomic position: a retrospective cohort study. Ann Intern Med. 2017;167(7):456-464. doi:10.7326/M16-2543 28847012 PMC6435027

[44] Pabayo R, Kawachi I, Gilman SEUS US state-level income inequality and risks of heart attack and coronary risk behaviors: longitudinal findings. Int J Public Health. 2015;60(5):573-588. doi:10.1007/s00038-015-0678-7 25981210 PMC4517572

[45] Strobl C, Malley J, Tutz G An introduction to recursive partitioning: rationale, application, and characteristics of classification and regression trees, bagging, and random forests. Psychol Methods. 2009;14(4):323-348. doi:10.1037/a0016973 19968396 PMC2927982

