# Supplementary material for: Value of Neighborhood Socioeconomic Status in Predicting Risk of Outcomes in Studies That Use Electronic Health Record Data
Source: JAMA Netw Open. 2018 Sep 21;1(5):e182716. doi: 10.1001/jamanetworkopen.2018.2716 (PMC6324505; doi:10.1001/jamanetworkopen.2018.2716)
Supplement: Supplement. — eTable 1. Demographic and Clinical Characteristics of Patients eTable 2. Model Fit Results Using Cross-Validation in the Training Data eTable 3. Model Fit Results Using Principal Components of ACS Data eFigure. Discrete Time Prediction Results [file jamanetwopen-1-e182716-s001.pdf]

Supplementary Online Content

Bhavsar NA, Gao A, Phelan M, Pagidipati NJ, Goldstein BA. Value of neighborhood socioeconomic status in predicting risk of outcomes in studies that use electronic health record data. *JAMA Netw Open*. 2018;1(5):e182716. doi:10.1001/jamanetworkopen.2018.2716

eTable 1. Demographic and Clinical Characteristics of Patients
eTable 2. Model Fit Results Using Cross-Validation in the Training Data
eTable 3. Model Fit Results Using Principal Components of ACS Data
eFigure. Discrete Time Prediction Results

This supplementary material has been provided by the authors to give readers additional information about their work.

© 2018 Bhavsar NA et al. *JAMA Network Open.*

eTable 1. Demographic and Clinical Characteristics of Patients

	Train	Test	SES Quartile1	SES Quartile 2	SES Quartile 3	SES Quartile 4
**N**	90097	122812	21906	21960	25071	21160
**Index**	56.97 (7.54)	56.84 (7.72)	46.63 (3.60)	54.65 (1.67)	60.56 (1.20)	65.81 (3.77)
**Age**	47.17 (17.71)	46.16 (17.94)	44.79 (17.96)	46.44 (17.85)	48.00 (17.56)	49.39 (17.14)
**Male Gender (%)**	32590 (36.2)	47295 (38.5)	7779 (35.5)	7561 (34.4)	9213 (36.7)	8037 (38.0)
**Race (%)**						
Black	37774 (41.9)	48766 (39.7)	16351 (74.6)	10624 (48.4)	6417 (25.6)	4382 (20.7)
Hispanic	3831 (4.3)	7528 (6.1)	1478 (6.7)	1430 (6.5)	600 (2.4)	323 (1.5)
White	42950 (47.7)	56321 (45.9)	3086 (14.1)	8686 (39.6)	16400 (65.4)	14778 (69.8)
Other	5542 (6.2)	10197 (8.3)	991 (4.5)	1220 (5.6)	1654 (6.6)	1677 (7.9)
**Primary Payer (%)**						
Private	53326 (59.2)	68473 (55.8)	7795 (35.6)	12332 (56.2)	17348 (69.2)	15851 (74.9)
Public	21566 (23.9)	28032 (22.8)	6719 (30.7)	5422 (24.7)	5291 (21.1)	4134 (19.5)
Self-pay	7031 (7.8)	13656 (11.1)	3203 (14.6)	1878 (8.6)	1249 (5.0)	701 (3.3)
Unknown	8174 (9.1)	12651 (10.3)	4189 (19.1)	2328 (10.6)	1183 (4.7)	474 (2.2)
**Service Utilization**						
ED visits	0.48 (1.46)	0.44 (1.37)	0.97 (2.14)	0.52 (1.51)	0.27 (0.95)	0.16 (0.66)
IP visits	0.14 (0.53)	0.11 (0.46)	0.20 (0.68)	0.14 (0.55)	0.11 (0.46)	0.09 (0.38)
OP visits	6.37 (8.86)	7.07 (10.10)	6.59 (9.22)	6.42 (9.17)	6.21 (8.64)	6.31 (8.40)
IP Length of Stay	0.64 (4.04)	0.54 (4.15)	0.98 (4.86)	0.69 (4.70)	0.51 (3.44)	0.40 (2.82)
Public Clinic Visit	11603 (12.9)	18160 (14.8)	6077 (27.7)	3336 (15.2)	1570 (6.3)	620 (2.9)
**Medication Use (%)**						
CCB	7941 (8.8)	13217 (10.8)	2659 (12.1)	1918 (8.7)	1875 (7.5)	1489 (7.0)
LOOP	3565 (4.0)	5264 (4.3)	1207 (5.5)	898 (4.1)	857 (3.4)	603 (2.8)
THZ	10365 (11.5)	15584 (12.7)	3048 (13.9)	2683 (12.2)	2505 (10.0)	2129 (10.1)
ARA	1180 (1.3)	2321 (1.9)	471 (2.2)	289 (1.3)	241 (1.0)	179 (0.8)
BB	11409 (12.7)	17151 (14.0)	3142 (14.3)	2771 (12.6)	3000 (12.0)	2496 (11.8)
STA	13297 (14.8)	21467 (17.5)	3244 (14.8)	3090 (14.1)	3695 (14.7)	3268 (15.4)
ACE	11411 (12.7)	17072 (13.9)	3354 (15.3)	2822 (12.9)	2888 (11.5)	2347 (11.1)

© 2018 Bhavsar NA et al. *JAMA Network Open.*

ARB	10182 (11.3)	17329 (14.1)	2309 (10.5)	2427 (11.1)	2812 (11.2)	2634 (12.4)
DIAB	6438 (7.1)	10728 (8.7)	2257 (10.3)	1648 (7.5)	1496 (6.0)	1037 (4.9)
**Medical History (%)**						
CVD	2398 (2.7)	2951 (2.4)	697 (3.2)	615 (2.8)	629 (2.5)	457 (2.2)
DIAB	9997 (11.1)	13394 (10.9)	3408 (15.6)	2637 (12.0)	2302 (9.2)	1650 (7.8)
Hypertension	25876 (28.7)	31929 (26.0)	7588 (34.6)	6547 (29.8)	6483 (25.9)	5258 (24.8)
COPD	2754 (3.1)	5003 (4.1)	844 (3.9)	713 (3.2)	727 (2.9)	470 (2.2)
CHF	2042 (2.3)	2889 (2.4)	734 (3.4)	504 (2.3)	468 (1.9)	336 (1.6)
PVD	1745 (1.9)	2415 (2.0)	517 (2.4)	439 (2.0)	456 (1.8)	333 (1.6)
CKD	6841 (7.6)	9287 (7.6)	2062 (9.4)	1753 (8.0)	1728 (6.9)	1298 (6.1)
MI	1194 (1.3)	1481 (1.2)	359 (1.6)	306 (1.4)	313 (1.2)	216 (1.0)
Stroke	1042 (1.2)	1291 (1.1)	302 (1.4)	275 (1.3)	269 (1.1)	196 (0.9)
**No. of Laboratory Test**						
SBP	1.51 (2.65)	2.22 (3.29)	1.88 (2.96)	1.56 (2.70)	1.35 (2.48)	1.27 (2.40)
DBP	1.51 (2.65)	2.22 (3.29)	1.88 (2.95)	1.56 (2.70)	1.35 (2.48)	1.27 (2.39)
Weight	1.45 (2.52)	2.16 (2.95)	1.86 (2.86)	1.51 (2.58)	1.27 (2.35)	1.17 (2.21)
Hb1AC	0.25 (0.77)	0.28 (0.77)	0.34 (0.92)	0.26 (0.79)	0.21 (0.70)	0.19 (0.65)
Creatine	1.50 (4.84)	2.11 (7.47)	2.05 (6.05)	1.57 (5.14)	1.29 (4.14)	1.11 (3.70)
Cholesterol	0.42 (0.68)	0.52 (0.93)	0.42 (0.71)	0.41 (0.68)	0.41 (0.67)	0.44 (0.68)
RG	3.12 (17.58)	2.88 (16.04)	4.75 (23.06)	3.33 (18.68)	2.47 (15.13)	1.99 (11.25)
HDL	0.41 (0.67)	0.51 (0.92)	0.41 (0.68)	0.41 (0.67)	0.40 (0.66)	0.44 (0.68)
LDL	0.41 (0.67)	0.51 (0.92)	0.40 (0.67)	0.40 (0.66)	0.40 (0.66)	0.43 (0.67)
MCHC	1.26 (4.51)	1.81 (6.94)	1.81 (5.74)	1.32 (4.67)	1.04 (3.90)	0.89 (3.38)
MCV	1.26 (4.51)	1.81 (6.95)	1.81 (5.74)	1.32 (4.67)	1.04 (3.90)	0.89 (3.38)
WBC	1.26 (4.51)	1.81 (6.93)	1.81 (5.74)	1.32 (4.67)	1.04 (3.90)	0.89 (3.37)
RDW	1.26 (4.50)	1.80 (6.92)	1.81 (5.74)	1.32 (4.66)	1.03 (3.88)	0.89 (3.38)
Sodium	1.51 (4.83)	2.12 (7.46)	2.03 (6.04)	1.56 (5.13)	1.30 (4.12)	1.17 (3.70)

© 2018 Bhavsar NA et al. *JAMA Network Open.*

eTable 2 – Model fit results using cross-validation in the training data

Table S2: Sensitivity Analysis Using PCA

Outcome	EHR	EHR and PCA	PCA
**Myocardial Infarction**	0.892	0.892	0.504
**Stroke**	0.854	0.844	0.479
**Asthma**	0.752	0.763	0.618
**Accident**	0.747	0.754	0.616
**Emergency Department Visit**	0.746	0.751	0.621
**Inpatient Visit**	0.740	0.736	0.521
**Outpatient Visit**	0.674	0.675	0.506
**Flu**	0.562	0.579	0.498

* Model fitting using the top Principal Components from the ACS data instead of just the nSES metric.

eTable 3 – Model fit results using principal components of ACS data

Table S3: Sensitivity Analysis Using Cross-validation

Outcome	EHR	EHR and nSES	nSES
**Myocardial Infarction**	0.879	0.879	0.536
**Stroke**	0.818	0.821	0.529
**Accident**	0.744	0.753	0.641
**Emergency Department Visit**	0.740	0.748	0.645
**Asthma**	0.731	0.735	0.613
**Inpatient Visit**	0.728	0.732	0.555
**Outpatient Visit**	0.668	0.677	0.505
**Flu**	0.562	0.567	0.506

* Model fit using cross-validation in the training data. Results are comparable to what was found in the test data.

© 2018 Bhavsar NA et al. *JAMA Network Open.*

eFigure – Discrete time prediction results

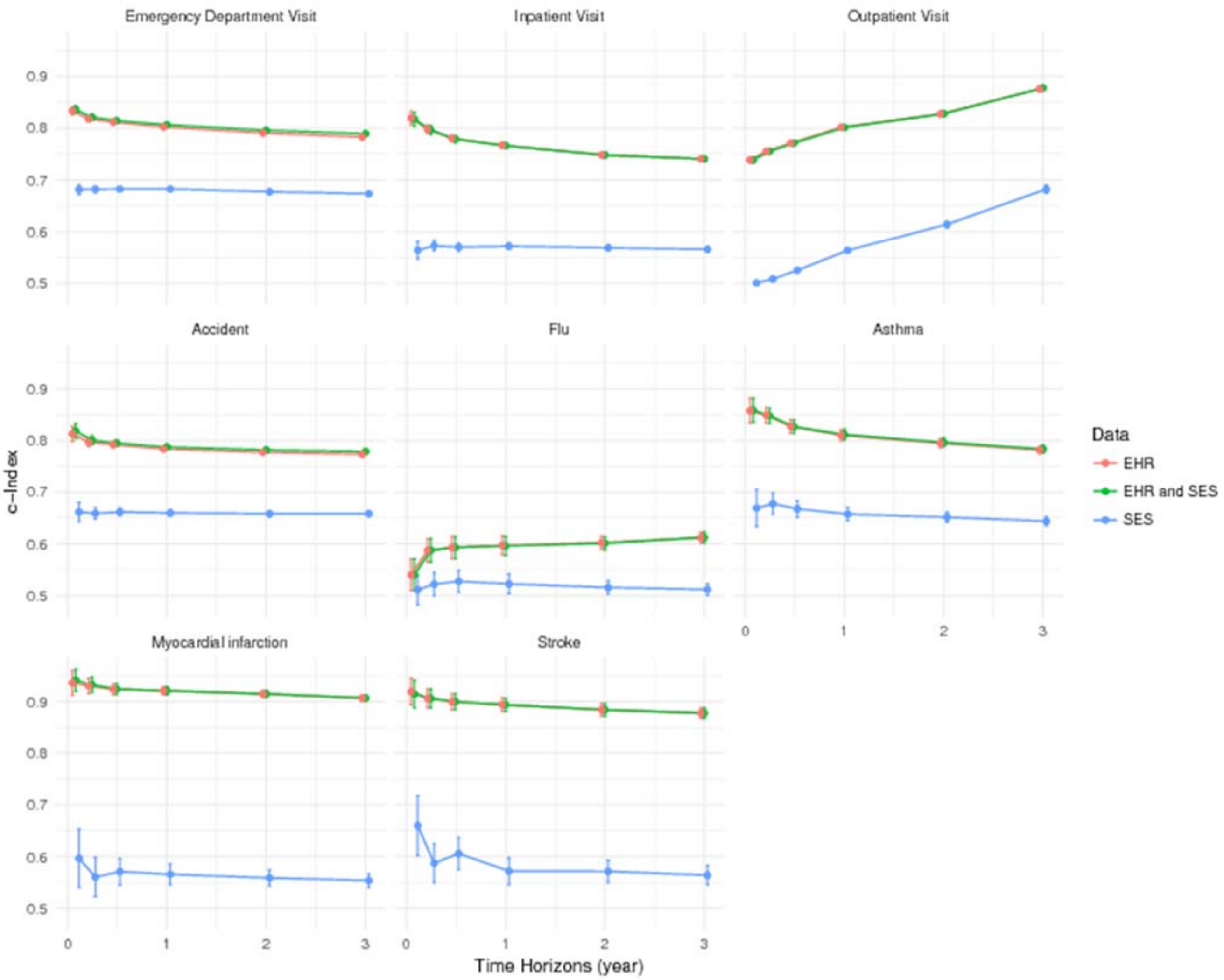

* Predictability at discrete time horizons. There is no specific trend towards nSES data being more predictive at farther time points.

© 2018 Bhavsar NA et al. *JAMA Network Open.*
